# Real‐world evidence supports the safety and efficacy profile of luspatercept in clinically complex and heavily iron‐overloaded patients

**DOI:** 10.1111/bjh.70437

**Published:** 2026-03-17

**Authors:** Barbara Gianesin, Antonietta Zappu, Giovanni Battista Ferrero, Carmen Maria Gaglioti, Michele Santodirocco, Giuseppe Fania, Giovanna Graziadei, Daniele Lello Panzieri, Antonella Massa, Tommaso Mina, Francesca Polese, Angelantonio Vitucci, Francesco Arcioni, Maria Caterina Putti, Valerio Cecinati, Ilaria Fotzi, Paola Maria Grazia Sanna, Antonio Cappello, Filomena Longo, Roberto Lisi, Annamaria Pasanisi, Daniela Pietrasanta, Monica Bocchia, Elisa Bertoni, Antonella Cossu, Marilena Serra, Susanna Barella, Antonia Gigante, Gian Luca Forni, Raffaella Origa

**Affiliations:** ^1^ FORANEMIA ETS Foundation Genoa Italy; ^2^ S.C. Centro delle Microcitemie e Anemie Rare, ARNAS G. Brotzu Cagliari Italy; ^3^ Thalassemia and Rare Hematological Diseases Centre San Luigi Gonzaga University Hospital Orbassano (Torino) Italy; ^4^ Day Hospital Thalassemia, IRCCS “Casa Sollievo della Sofferenza” San Giovanni Rotondo (FG) Italy; ^5^ Immunoematologia e Medicina Trasfusionale (SIMT), IRCCS “Casa Sollievo della Sofferenza” San Giovanni Rotondo (FG) Italy; ^6^ Fondazione IRCCS Ca' Granda Ospedale Maggiore Policlinico, SC Medicina ad Indirizzo Metabolico Milan Italy; ^7^ Università degli Studi di Milano Milan Italy; ^8^ Servizio Trasfusionale, Ospedale “Giovanni Paolo II” Olbia Italy; ^9^ Pediatric Hematology/Oncology, Fondazione IRCCS Policlinico San Matteo Pavia Italy; ^10^ U.O. Medicina Trasfusionale, Ospedale dell'Angelo, Azienda ULSS 3 Serenissima Mestre Venice Italy; ^11^ Hematology and Bone Marrow Transplantation Unit, AOUC Policlinico Bari Italy; ^12^ Pediatric Oncology‐Hematology Unit, Santa Maria della Misericordia Hospital Perugia Italy; ^13^ Clinic of Pediatric Hematology Oncology, Department of Women's and Children's Health University Hospital of Padua Padua Italy; ^14^ U.O.C. di Pediatria Oncoematologia Pediatrica e Microcitemia Ospedale Santissima Annunziata Taranto Italy; ^15^ U.O.C. “Oncologia, Ematologia e Trapianto di Cellule Staminali Emopoietiche” Meyer Children's Hospital IRCCS Florence Italy; ^16^ Servizio Trasfusionale Aziendale, Azienda Ospedaliero‐Universitaria di Sassari Sassari Italy; ^17^ ASL Novara—Ospedale Ss. Trinità Borgomanero Novara Italy; ^18^ U.O.C. Talassemie ed Emoglobinopatie, Azienda Ospedaliero Universitaria S. Anna Ferrara Italy; ^19^ Thalassemia Unit, ARNAS Garibaldi Catania Italy; ^20^ U.O.S. Centro Microcitemia “A. Quarta”—U.O.C. Ematologia PO Perrino Brindisi Italy; ^21^ Division of Hematology Azienda Ospedaliera SS Antonio e Biagio e Cesare Arrigo Alessandria Italy; ^22^ Department of Medical Science, Surgery and Neuroscience Hematology, University of Siena Siena Italy; ^23^ U.O. Oncoematologia Pediatrica e Trapianto di Midollo Osseo, ASST Spedali Civili di Brescia Brescia Italy; ^24^ Centro Microcitemie, Presidio Ospedaliero San Francesco Nuoro Italy; ^25^ Internal Medicine Unit, Thalassemia Centre, “Fazzi” Hospital Lecce Italy; ^26^ Haematology Unit IRCCS Giannina Gaslini Genoa Italy; ^27^ Department of Medical Sciences and Public Health University of Cagliari Cagliari Italy

**Keywords:** extramedullary haematopoiesis, luspatercept, real‐world setting, thromboembolism, transfusion‐dependent thalassaemia

To the Editor,

Luspatercept has been demonstrated to effectively reduce transfusion requirements in adult patients with transfusion‐dependent β‐thalassaemia (TDT), as shown in the phase 3 BELIEVE trial and its extension study.^1^, ^2^ Following several heterogeneous and generally small real‐world reports,^3^, ^4^, ^5^, ^6^ a recent multicentre Italian study involving 231 TDT patients—the largest cohort reported to date—has further confirmed the efficacy of luspatercept in everyday clinical care.^7^ Moreover, that study—including patients who started luspatercept treatment after the marketing authorization in Italy—provided a detailed characterization of the drug's safety profile in real‐world conditions.^8^

Conversely, a group of Italian patients with TDT were granted the opportunity—following approval by the local Ethics Committee for each individual case—to initiate luspatercept during the *compassionate use* phase, as early access to the drug was deemed a priority and potentially a life‐saving therapy, prior to marketing authorization.

Among these patients, 111 individuals from 22 centres within the Italian Society of Thalassemia and Hemoglobinopathies network provided written informed consent for the collection of safety and efficacy data on the *compassionate use* phase and a maximum of 24 months after the drug became available through the National Health System. Of these, 54 (48.6%) were male, and the mean age ± standard deviation (SD) at treatment initiation was 40.3 ± 9.9 years. Sixty‐one patients (55.0%) had a *β* ^0^/*β* ^0^ genotype, and 37 (33.3%) had undergone splenectomy (Table 1).

**TABLE 1:** Baseline comorbidities in the studied cohort.

Variable	Value
Number of patients (% males)	111 (49%)
Genotype distribution—*n* (%)	
*β* ^0^/*β* ^0^	61 (55.0%)
*β* ^0^/*β* ^+^	37 (33.3%)
*β* ^+^/*β* ^+^	2 (1.8%)
*β* ^0^/*β* ^wt^ + *α* triplication or quadruplication	3 (2.7%)
*HbE/β+*	1 (0.9)
NA	7 (6.3%)
Age (years) at baseline	42 (33–47)
Age (years) at diagnosis	0.8 (0–1)
Age (years) at first transfusion	1 (0.5–2)
Ferritin (ng/mL)	931 (439–2142)
Ferritin ≥1000 ng/mL (%)	51/109 (46.8%)
Ferritin ≥2500 ng/mL (%)	23/109 (21.1%)
Liver iron concentration (LIC) (mg Fe/g dry weight)	3.76 (2.17–9.97)
LIC ≥3 mg Fe/g dry weight (%)	59/96 (61.5%)
LIC ≥7 mg Fe/g dry weight (%)	31/96 (32.3%)
LIC ≥15 mg Fe/g dry weight (%)	19/96 (19.8%)
MRI‐T2^a^ (ms)	35.0 (24.2–40.6)
MRI‐T2^a^ ≤ 20 ms (%)	19/98 (19.4%)
Iron overload^b^ (%)	61/110 (55.5%)
Transfusion: 12 weeks before start of therapy	
Units transfused	10 (8–12)
Pure RBC transfused (mL)	1649 (1303–1930)
Pre‐transfusion Hb	9.5 (9.2–10.1)
Transfusion: 24 weeks before start of therapy	
Units transfused	20 (14–24)
Pure RBC transfused (mL)	3300 (2412–3805)
Pre‐transfusion Hb	9.6 (9.2–10)
Red blood cell units in the 24 weeks prior to baseline	
6–20 units (%)	63 (56.8%)
21–24 units (%)	32 (28.8%)
25–32 units (%)	16 (14.4%)
Medical history	
Comorbidities number/patient	
Mean (SD)	3.7 (2.3)
Median (Q1–Q3)	3 (2–5)
Min–max	0–10
Number of patients with at least one comorbidity (%)	107/111 (96.4%)
Masses of extramedullary erythropoiesis (%)	11/89 (12.4%)
Smoke (%)	19/110 (17.3%)
Obesity (%)	6/111 (5.4%)
Oestrogen–progestin therapy (%)	22/109 (20.2%)
Testosterone therapy (%)	17/109 (15.6%)
Regular menstrual cycle (%)	31/54 (57.4%)
Congenital thrombophilia (%)	3/98 (3.1%)
Autoimmune haemolytic anaemia (%)	3/111 (2.7%)
History of deep vein thrombosis (%)	2/111 (1.8%)
Family history of thrombosis (%)	6/103 (5.8%)
History of arterial thrombosis (%)	0/111
History of stroke (%)	1/111 (0.9%)
History of TIA (transient ischaemic attack) (%)	1/111 (0.9%)
Chronic kidney disease (%)	3/111 (2.7%)
Adrenal insufficiency (%)	1/111 (0.9%)
Hypoparathyroidism (%)	7/110 (6.4%)
Hypothyroidism (%)	20/111 (18.0%)
Hypogonadism (%)	41/111 (36.9%)
Diabetes (%)	14/111 (12.6%)
Splenectomy (%)	37/111 (33.3%)
Neoplasm (%)	2/111 (1.8%)
History of hepatitis B virus infection (%)	9/111 (8.18%)
History of hepatitis C virus infection (%)	47/111 (42. 3%)
Cirrhosis (%)	0/111
Pulmonary hypertension (requiring therapy) (%)	1/111 (0.9%)
QT prolongation	0/105
Atrial flutter/atrial fibrillation (%)	8/110 (7.34%)
No sustained ventricular tachycardia/other clinically significant arrhythmia (%)	5/110 (4.6%)
History of acute myocardial infarction (%)	0/111 (0%)
Left ventricular ejection fraction (LVEF) below 56% (%)	16/111 (14.4%)
Heart failure (%)	12/111 (10.8%)
History of sepsis (%)	8/111 (7.2%)
Iron chelators, *n* (%)	
DFP	18/110 (16.4%)
DFX	58/110 (52.7%)
DFO	8/110 (7.3%)
DFO + DFP	5/110 (4.5%)
DFO + DFX	8/110 (7.3%)
DFX + DFP	13/110 (11.8%)
Compliance with iron chelation treatment	
Good (≥80%)	72/109 (66.1%)
Medium (≥50 to <80%)	20/109 (18.3%)
Poor (<50%)	17/109 (16.6%)

*Note*: All values are reported as median and interquartile range (Q1–Q3: 25th–75th interquartile). Percentages are calculated on available data.

Abbreviations: DFO, deferoxamine; DFP, deferiprone; DFX, deferasirox; Hb, haemoglobin; MRI, magnetic resonance imaging; RBC, red blood cell; SD, standard deviation.

^a^ Significance of chi‐squared test/Fisher exact test, Wilcoxon signed‐rank test for number of comorbidities.

^b^ Ferritin ≥2500 ng/mL and/or LIC ≥3 mg Fe/g dry weight and/or MRI T2 ≤ 20 ms.

As expected from the definition of *compassionate use*, this cohort showed a more severe clinical phenotype than patients from the recent Italian real‐world study, with a higher burden of comorbidities in the *compassionate use* cohort (3.7 ± 2.3 vs. 3.0 ± 2.0; *p* = 0.009) as well as a significantly increased likelihood of cardiac involvement.^1^, ^2^, ^7^ Sixteen patients (14.4%) had a left ventricular ejection fraction (LVEF) below 56% at treatment initiation, and 12 (10.8%) had a documented history of heart failure. In addition, these patients exhibited a greater transfusion burden (Table S1).

Iron overload was also significantly greater than that reported by Origa et al.^7^ (Table S2). The median serum ferritin (25th–75th interquartile, Q1–Q3) prior to treatment was 931 ng/mL (439–2142), with 21.1% of patients exceeding 2500 ng/mL. The median liver iron concentration (LIC) was 3.76 mg/g dw (2.17–9.97), and 19.8% of patients exhibited severe hepatic iron overload (LIC ≥ 15 mg/g dw). In addition, 19.4% had cardiac T2* values ≤20 ms, consistent with cardiac iron accumulation. In line with these findings, 26 patients (23.6%) were receiving intensive chelation therapy with the association of two iron chelators.

The mean duration of treatment was 90 ± 66 weeks (range 3–240), and 72 (64.9%) patients continued to receive luspatercept after its marketing authorization. The discontinuation rate within the first 24 weeks was 21.6%, which was significantly higher than that reported in the BELIEVE trial (*6.25%*; *p* < 0.001),^1^, ^2^ but comparable to that observed among Italian patients who initiated therapy after commercial availability (*25.1%*; *p* = 0.56).^7^ At the time of observation, 66.9% of the patients had stopped the treatment.

An unexpected pregnancy, subsequently resulting in a healthy newborn, led to the discontinuation of treatment in one patient.^9^ Therapy was discontinued by 30 patients due to lack of efficacy and 33 due to adverse events (Table S3 and Figure S1). Additionally, four patients withdrew due to logistical complexities, two for personal reasons and two died from causes unrelated to luspatercept (sudden at‐home deaths linked to severe pre‐existing cardiac disease).

These findings underscore that luspatercept treatment is a complex therapeutic approach requiring a clear discussion of expectations between physician and patient prior to initiation as well as a continuous strengthening of the therapeutic alliance over time. This is particularly relevant given that the drug directly affects an essential and deeply personal aspect of patient care (transfusion regimen and frequency), which often carries profound psychological implications.^6^ It should also be noted that, owing to the presence of multiple comorbidities and suboptimal iron overload control, these patients may be considered clinically challenging to manage, which could have contributed, at least in part, to the high rate of early discontinuation.

In further support of this finding, adherence to iron chelation therapy during luspatercept treatment was reported as good in 66.1%, moderate in 18.3% and poor in 15.6% of patients—an observation that clearly influences outcomes related to iron burden.

Unfortunately, the number of patients with serial magnetic resonance imaging (MRI) assessments was insufficient to draw definitive conclusions. Analysis of ferritin trends over time, however, demonstrated a significant decrease (last vs. baseline evaluation, 732 ng/mL Q1–Q3: 329–1611 vs. 931 ng/mL Q1–Q3: 439–2142, *p* = 0.004) with no difference between responders and non‐responders. Nevertheless, ferritin variability was higher than that observed by Origa et al.,^7^ with a coefficient of variation 1.8 times greater in the *compassionate use* cohort (*p* < 0.001).

Chelation therapy was modified in 31 patients: 18 had dose adjustments, 9 changed chelator and 4 had both. Dose reductions were mainly prompted by decreases in ferritin levels (Table S4). Clinically, a drop in ferritin levels frequently triggers a further decline in chelation adherence among patients with a history of poor compliance. However, regardless of luspatercept's potential effects on iron metabolism, iron stores cannot decrease in the absence of adequate chelation. For this reason, chelation therapy should not be adjusted solely based on ferritin trends, but rather in the context of both changes in transfusion requirements and organ iron load as assessed by MRI.

Despite the overall more severe clinical phenotype of this cohort, the effect of luspatercept on transfusion requirements remained statistically significant, with results at least comparable to those reported in both the Italian real‐world experience and/or the BELIEVE trial^1^, ^2^, ^7^ (Figure 1).

**FIGURE 1:**
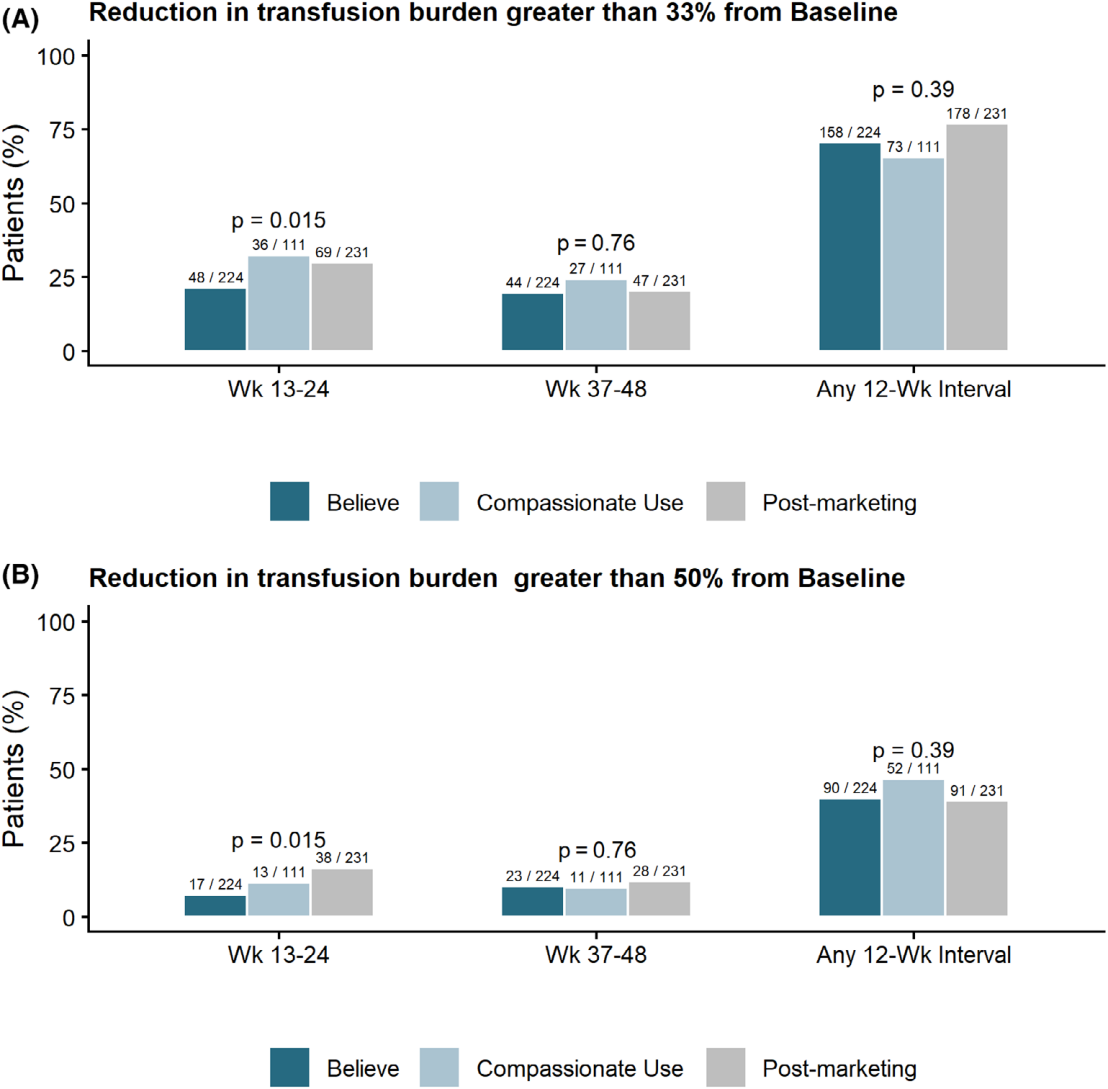
Comparison among the three luspatercept cohorts: BELIEVE cohort,^1^ *Compassionate use* cohort and post‐marketing cohort.^3^

Fourteen patients reached transfusion independence (median value of the maximum transfusion interval for each patient 19.9 weeks, Q1–Q3: 9.25–28.4). At univariate analysis, factors associated with transfusion independence were a non‐*β* ^0^/*β* ^0^ genotype (12/14 vs. 18/68, *p* < 0.001), a lower baseline transfusion burden (7.5 units Q1–Q3: 8–12 vs. 11 units Q1–Q3: 6–10, *p* = 0.0015) and a lower value of serum ferritin level (501 ng/mL, Q1–Q3: 337–1165 vs 1178 ng/mL, Q1–Q3: 694–2750, *p* = 0.031), all features typically observed in patients with non‐transfusion‐dependent thalassaemia. Upon multivariate analysis, the final model included only genotype (*p* = 0.001) and transfusion burden (*p* = 0.006) (Table S5).

Overall, 36 patients (32.4%) achieved a ≥33% reduction in transfused blood units between weeks 13 and 24 (Figure 1), while 13 patients (11.7%) achieved a ≥50% reduction during the same period. When considering any 12‐week interval throughout treatment, 73 patients (65.8%) and 52 patients (46.8%) achieved a ≥33% and ≥50% reduction, respectively, in transfusion burden.

Consistent with these findings, although pre‐transfusion haemoglobin (Hb) levels did not increase—neither in the overall cohort nor in the subgroup of patients with baseline Hb <9.5 g/dL—the efficacy according to the classification of Musallam et al. was similar to that already reported.^7^, ^10^ Specifically, 9 (11.0%) patients demonstrated an excellent response, 46 (56.1%) a good response, 9 (11.0%) a sufficient response and 18 (22.0%) no response.

The apparent lack of a rise in pre‐transfusion Hb values (Figure S2) may be explained by the early stage of the *compassionate use* experience and the clinicians' tendency to adhere to the trial protocol requirements by maintaining the same pre‐transfusion Hb target as in the pretreatment phase, in order to properly assess treatment efficacy.^11^ However, the absence of a substantial rise in pre‐transfusion Hb does not preclude treatment efficacy, as the primary clinical benefit of this agent lies in reducing transfusion dependence.

The most frequently reported adverse events were consistent with previously published data, with bone pain being the most common—typically occurring during the initial treatment cycles—followed by fatigue and arthralgia (Table S3 and Figure S1).^1^, ^2^, ^7^

Thrombotic events were observed in three patients, corresponding to a percentage consistent with that of previous studies.^1^, ^7^ Two patients experienced deep vein thrombosis complicated with pulmonary embolism, while one patient developed superficial vein thrombosis. One of the two patients who experienced deep vein thrombosis had not undergone splenectomy (Table S6). All patients had additional risk factors beyond thalassaemia itself. Given the potential contributory role of luspatercept, these data confirm the need for careful patient selection before initiating therapy.

There was one case of new appearance of extramedullary erythropoietic masses (EMH) and one case of enlargement. The prevalence may be underestimated due to the lack of shared recommendations regarding mass identification at treatment initiation and follow‐up at the start of *compassionate use* phase (Table S7).

A total of 25 hospitalizations occurred in 17 patients (6 patients with two admissions, 1 with three) (Table S8). The primary cause was cardiac (12 cases), including three episodes of heart failure (one with reduced LVEF), six episodes of atrial fibrillation (one of new onset) and one episode of atrial fibrillation associated with heart failure (Table S7).

In conclusion, the safety and efficacy profile of luspatercept is confirmed in real‐world practice in the Italian cohort of patients enrolled for the early access programme, which included patients with a high transfusion burden, a great number of comorbidities and also a substantial iron overload.

## AUTHOR CONTRIBUTIONS

Conceptualization and methodology: Raffaella Origa and Barbara Gianesin. Data curation, formal analysis and visualization: Barbara Gianesin. Investigation: Antonietta Zappu, Giovanni Battista Ferrero, Carmen Maria Gaglioti, Michele Santodirocco, Giuseppe Fania, Giovanna Graziadei, Daniele Lello Panzieri, Antonella Massa, Tommaso Mina, Francesca Polese, Angelantonio Vitucci, Francesco Arcioni, Maria Caterina Putti, Valerio Cecinati, Ilaria Fotzi, Paola Maria Grazia Sanna, Antonio Cappello, Filomena Longo, Roberto Lisi, Annamaria Pasanisi, Daniela Pietrasanta, Monica Bocchia, Elisa Bertoni, Antonella Cossu, Marilena Serra, Susanna Barella, Raffaella Origa. Supervision: Gian Luca Forni, Raffaella Origa. Project administration: Barbara Gianesin, Antonietta Zappu, Antonia Gigante, Raffaella Origa. Funding acquisition: Antonia Gigante, Gian Luca Forni. Writing—original draft: Barbara Gianesin, Raffaella Origa. Writing—review & editing: Barbara Gianesin, Antonietta Zappu, Giovanni Battista Ferrero, Carmen Maria Gaglioti, Michele Santodirocco, Giuseppe Fania, Giovanna Graziadei, Daniele Lello Panzieri, Antonella Massa, Tommaso Mina, Francesca Polese, Francesco Arcioni, Maria Caterina Putti, Valerio Cecinati, Ilaria Fotzi, Paola Maria Grazia Sanna, Antonio Cappello, Filomena Longo, Roberto Lisi, Annamaria Pasanisi, Daniela Pietrasanta, Monica Bocchia, Elisa Bertoni, Antonella Cossu, Marilena Serra, Susanna Barella, Antonia Gigante, Gian Luca Forni, Raffaella Origa.

## FUNDING INFORMATION

This non‐profit research was carried out with an unrestricted grant from Bristol‐Myers Squibb Services Unlimited. The sponsor had no influence on the study design, data collection, analysis, interpretation or manuscript preparation.

## CONFLICT OF INTEREST STATEMENT

RO received speaker honoraria from BMS, Vertex and Chiesi and consultancy fees from BMS, Agios and Avanzanite Bioscience BV. GG has received speaker honoraria from Vertex and consultancy fees from BMS, Pfizer and Alnylam. FL received consultancy fees and speaker honoraria from BMS and Vertex Pharmaceuticals, consultancy fees from Agios Pharmaceuticals and Avanzanite Bioscience BV. The other authors declare that they have no conflicts of interest.

## Supporting information


Figure S1.
Figure S2.
Table S1.
Table S2.
Table S3.
Table S4.
Table S5.
Table S6.

## Data Availability

The datasets generated and analysed during the current study can be obtained from the corresponding author upon reasonable request.
