# Supplementary material for: Real‐world evidence supports the safety and efficacy profile of luspatercept in clinically complex and heavily iron‐overloaded patients
Source: Br J Haematol. 2026 Mar 17;208(6):2320–5. doi: 10.1111/bjh.70437 (PMC13267453; doi:10.1111/bjh.70437)
Supplement: Supplementary file 1 — Figure S1. Figure S2. Table S1. Table S2. Table S3. Table S4. Table S5. Table S6. [file BJH-208-2320-s001.docx]

**Supplementary Material: Methods**

Demographic, clinical, and laboratory data, as well as details on transfusions and luspatercept administration, were collected and managed using the electronic data capture tool REDCap (Research Electronic Data Capture) hosted on the server of the FORANEMIA ETS Foundation (https://redcap.foranemia.org/redcap/). Details on transfusions and luspatercept administration were collected from the first administration of luspatercept till the last follow-up or interruption of therapy. Laboratory parameters were collected at baseline, in the period of pre-commercial use of luspatercept, and at intervals of 12 weeks in the subsequent period. Adverse events as well hospitalizations were registered during the entire follow-up period. Compliance to iron chelation therapy was defined as good if greater than 80%, medium if between 50-80 % and poor if less than 50%. It was assessed based on the amount of medication dispensed by the pharmacy and/or on physician–patient interviews.

The 33% and 50% reductions in transfusion burden from baseline were calculated at weeks 13–24, and in any of the follow-up periods of 12 weeks, according to the pivotal Phase III study.^1^ Response to luspatercept was defined as excellent, good, and satisfactory, according to the definitions of luspatercept treatment efficacy by Musallam et al. (2023)^10^, excluding the impact of the drug on the quality of life due to a lack of data^7^:

- Excellent: (i) A persistent reduction of ≥50% in transfusion requirements within 6 months of therapy, with the same or a higher pre-transfusion Hb level; (ii) A persistent increase of ≥2 g/dL in pre-transfusion Hb levels, previously suboptimal, with the same or a lower transfusion regimen.
- Good: (i) A reduction of ≥33% in transfusion requirements within 6 months of therapy, with the same or a higher pre-transfusion Hb level; (ii) An increase of ≥1 g/dL in pre-transfusion Hb levels, previously suboptimal, with the same or a lower transfusion regimen.
- Satisfactory: Any reduction in transfusion requirements within 6 months of therapy, with the same or a higher pre-transfusion Hb level.
- No efficacy: All other cases.

A patient was defined transfusion independent in case of at least one transfusion interval greater than 8 weeks.

Descriptive analyses were performed using the counts and percentages for categorical and dichotomous variables. Continuous variables were described as median and interquartile range (Q1-Q3: 25^th^–75^th^interquartile) or mean ± standard deviation (SD). Groups were compared using the paired t-test or the Wilcoxon test, depending on the normality of distributions which was tested using the Shapiro–Wilk test. In case of multiple comparisons, the Holm multiple testing corrections were used to adjust p-values. The chi-square test or Fisher exact test was used to test the equality of proportions, Wilcoxon signed-rank test for multiple categories.

Univariate logistic regression analysis was used to study the association between the responders/non-responders and covariates at baseline. Multivariate logistic regression model was generated using a stepwise forward regression approach and considering variables with an overall percentage of completion >90%, and a level of significance p <0.20 at the univariate analysis.

For time-repeated measurements, a linear mixed model with a random intercept for each participant was used to evaluate the association between time and covariate (for example ferritin).

All statistical analyses were performed using the R version 4.2.2 (R Core Team, 2022). For all analyses, a p-value < 0.05 was considered statistically significant.

**Supplementary Material: Figure and Tables**


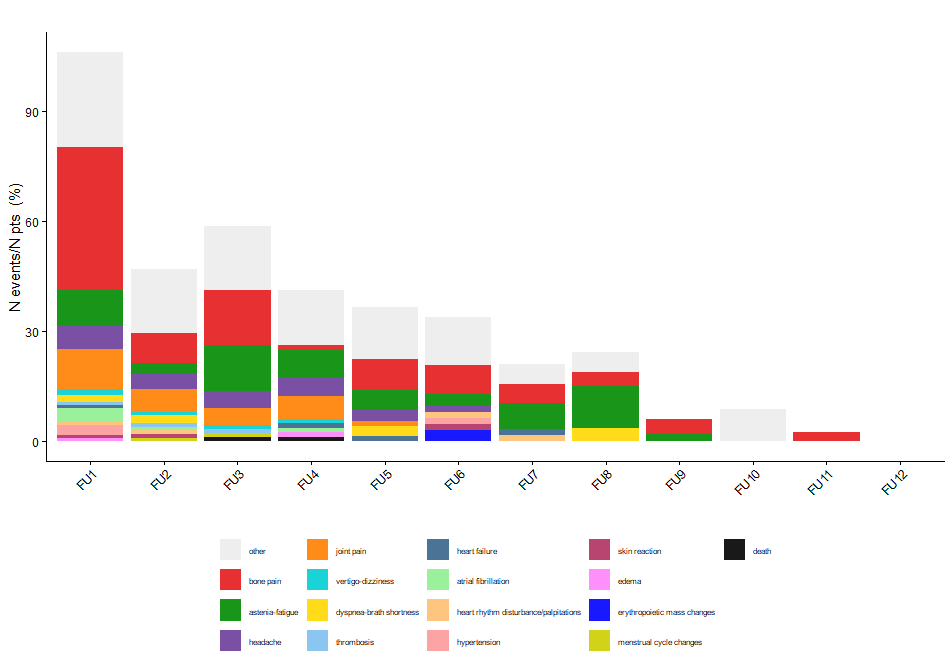


**Figure S1** Adverse events during the first 12 follow-ups of 12 weeks. Percentages were calculated considering the number of patients in each period of observation. Multiple events were considered for each patient.


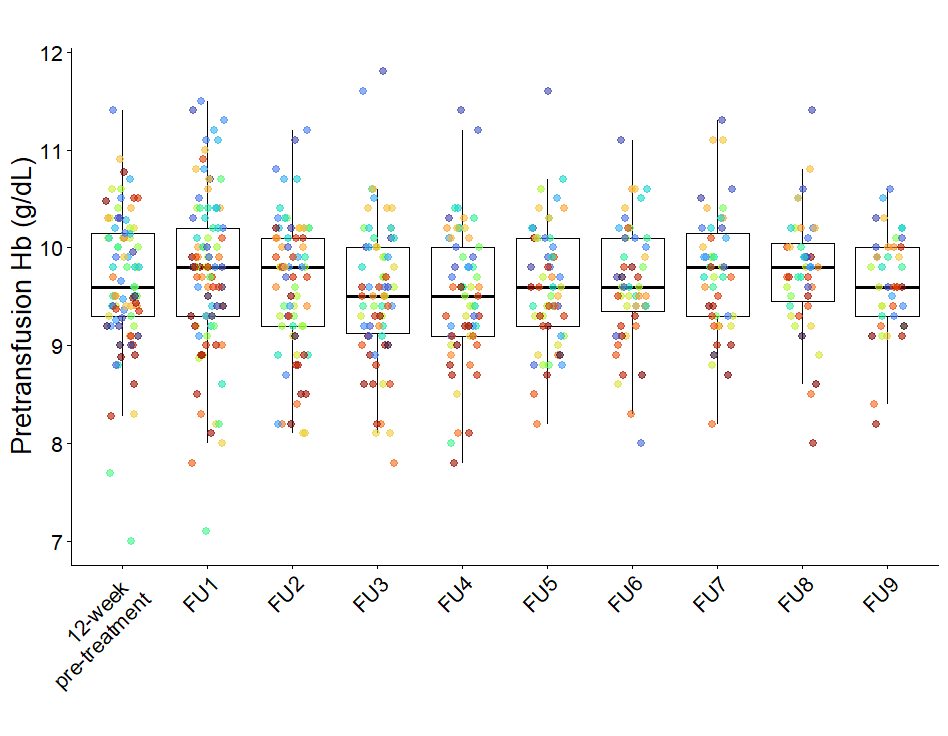


**Figure S2** Pretransfusion hemoglobin (Hb) values during the first 9 follow-ups after the start of therapy.

**Table S1** Baseline comorbidities in the studied cohort and comparison with the cohort which started luspatercept post-marketing^7^

|  | **Patients who started luspatercept in the ‘compassionate’ phase(n=111)** | **Patients who started luspatercept post-marketing**  **(n=231)** | **p-value*** |
| --- | --- | --- | --- |
| 12weeks- Red Blood Cells units at basal  Mean (SD)  Median (Q1-Q3)  Min-max | 10.3 (2.8)  10 (8-12)  4-16 | 9.3 (2.9)  9 (8-11)  8-18 | 0.003 |
| Comorbidities Number/patient  Mean (SD)  Median (Q1-Q3)  Min-max | 3.7 (2.3)  3 (2-5)  0-10 | 3.0 (2.0)  3 (2-4)  0-9 | 0.009 |
| Number of patients with at least one comorbidity (%) | 107/111 (96.4%) | 216/231 (93.5%) | 0.4 |
| Masses of extramedullary erythropoiesis (%) | 11/89 (12.4%) | 26/168 (15.5%) | 0.62 |
| Smoke (%) | 19/110 (17.3%) | 28/201 (14%) | 0.53 |
| Obesity (%) | 6/111 (5.4%) | 9/230 (3.9%) | 0.58 |
| Estrogen-progestin therapy (%) | 22/109 (20.2%) | 28/225 (12.4%) | 0.09 |
| Testosterone therapy (%) | 17/109 (15.6%) | 24/226 (10.6%) | 0.26 |
| Regular menstrual cycle (%) | 31/54 (57.4%) | 63/110 (57.3%) | 1 |
| Congenital thrombophilia (%) | 3/98 (3.1%) | 1/160 (0.6%) | 0.15 |
| Autoimmune hemolytic anemia (%) | 3/111 (2.7%) | 4/229 (1.7%) | 0.69 |
| History of deep vein thrombosis (%) | 2/111 (1.8%) | 4/227 (1.8%) | 1 |
| Family history of thrombosis (%) | 6/103 (5.8%) | 0/173 | 0.0024 |
| History of arterial thrombosis (%) | 0/111 | 1/225 (0.4%) | 1 |
| History of stroke (%) | 1/111 (0.9%) | 0/225 | 0.33 |
| History of TIA (transient ischemic attack)) (%) | 1/111 (0.9%) | 1/225 (0.4%) | 0.55 |
| Chronic kidney disease (%) | 3/111 (2.7%) | 2/229 (0.87%) | 0.33 |
| Adrenal insufficiency (%) | 1/111 (0.9%) | 3/229 (1.3%) | 1 |
| Hypoparathyroidism (%) | 7/110 (6.4%) | 9/230 (3.9%) | 0.46 |
| Hypothyroidism (%) | 20/111 (18.0%) | 46/230 (20%) | 0.77 |
| Hypogonadism (%) | 41/111 (36.9%) | 78/228 (34.2%) | 0.71 |
| Diabetes (%) | 14/111 (12.6%) | 25/230 (10.9%) | 0.77 |
| Splenectomy (%) | 37/111 (33.3%) | 98/230 (42.6%) | 0.13 |
| Neoplasm (%) | 2/111 (1.8%) | 2/228 (0.9%) | 0.6 |
| History of Hepatitis B virus infection (%) | 9/111 (8.18%) | 20/217 (9.2%) | 0.9 |
| History of Hepatitis C virus infection (%) | 47/111 (42.3%) | 103/228 (45.2%) | 0.71 |
| Cirrhosis (%) | 0/111 | 0/223 | 1 |
| Pulmonary hypertension (requiring therapy) (%) | 1/111 (0.9%) | 3/228 (1.3%) | 1 |
| QT prolongation | 0/105 | 0/218 | 1 |
| Atrial flutter / atrial fibrillation (%) | 8/110 (7.34%) | 19/229 (8.3%) | 0.91 |
| No sustained ventricular tachycardia / other clinically significant arrhythmia (%) | 5/110 (4.6%) | 9/230 (3.9%) | 0.78 |
| History of acute myocardial infarction (%) | 0/111 (0%) | 2/229 (0.87%) | 1 |
| Left ventricular ejection fraction (LVEF) below 56% (%) | 16/111 (14.4%) | 15/225 (6.7%) | 0.035 |
| Heart failure (%) | 12/111 (10.8%) | 8/229 (3.5%) | 0.015 |
| History of sepsis (%) | 8/111 (7.2%) | 2/97 (2.1%) | 0.11 |
| Percentages are calculated on available data.  *Significance of chi-square test/Fisher exact test, Wilcoxon signed-rank test for number of comorbidities. | | | |

**Table S2** Baseline iron overload status in the studied cohort and comparison with the cohort which started luspatercept post-marketing^7^

|  | **Patients who started luspatercept in the ‘compassionate’ phase**  **(n=111)** | **Patients who started luspatercept post-marketing**  **(n=231)** | **pvalue*** |
| --- | --- | --- | --- |
| Ferritin(ng/mL) | 931 (439- 2142) | 589 (327-995) | <0.0001 |
| Ferritin ≥ 1000 ng/mL (%) | 51/109(46.8%) | 53/219 (24.2%) | <0.0001 |
| Ferritin ≥ 2500 ng/mL (%) | 23/109 (21.1%) | 15/219 (6.8%) | 0.0003 |
| Liver Iron Concentration (LIC)mg Fe/g dry weight | 3.76 (2.17-9.97) | 2.19 (1.47-5.02) | <0.0001 |
| LIC ≥ 3 mg Fe/g dry weight (%) | 59/96 (61.5%) | 59/156 (37.8%) | 0.0004 |
| LIC ≥ 7 mg Fe/g dry weight (%) | 31/96 (32.3%) | 23/156 (14.7%) | 0.0016 |
| LIC ≥ 15 mg Fe/g dry weight (%) | 19/96 (19.8%) | 6/156 (3.8%) | <0.0001 |
| MRI-T2* (ms) | 35.0 (24.2-40.6) | 36.0 (29.0-41.5) | 0.11 |
| MRI-T2*≤ 20ms (%) | 19/98 (19.4%) | 6/159 (3.8%) | 0.00010 |
| Iron overload** (%) | 61/110 (55.5%) | 68/225 (30.2%) | <0.0001 |
| All values are reported asmedian and interquartile range (Q1-Q3: 25^th^–75^th^ interquartile). Percentages are calculated on available data.  *Significance of chi-square test/Fisher exact test, Wilcoxon signed-rank test for number of comorbidities.  **Ferritin ≥ 2500 ng/mL OR LIC ≥ 3 mg Fe/g dry weight OR MRI T2 ≤ 20 ms | | | |

**Table S3** Adverse events occurred in the first 12 follow-ups of 12 weeks.

| **Adverse event type (n=336)** | **Cases (n)** | **Percentage* (%)** | **Cause of hospitalization^§^** |
| --- | --- | --- | --- |
| **Bone pain** | 86 | 25.6 |  |
| **Astenia-fatigue** | 51 | 15.2 |  |
| **Joint pain** | 29 | 8.6 |  |
| **Headache** | 22 | 6.5 |  |
| **Atrial fibrillation/Heart rhythm disturbance/palpitations** | 10 | 3.0 | YES (4 pts) |
| **Dyspnea-breath shortness** | 8 | 2.4 |  |
| **Vertigo-dizziness** | 5 | 1.5 |  |
| **Hypertension** | 4 | 1.2 |  |
| **Heart failure** | 4 | 1.2 | YES (4 pt) |
| **Skin reaction** | 3 | 0.9 |  |
| **Thrombosis** | 3 | 0.9 |  |
| **Edema** | 2 | 0.6 |  |
| **Death** | 2 | 0.6 |  |
| **Erythropoietic mass changes** | 2 | 0.6 |  |
| **Menstrualcyclechanges** | 2 | 0.6 |  |
| **Other** | 103 | 30.7 | YES (10 pts) |
| Upper respiratory tract infection | 24 | 7.1 |  |
| Insomnia | 8 | 2.4 |  |
| Reaction to DFO | 7 | 2.1 |  |
| Epigastric pain | 6 | 1.8 |  |
| Depression–anxiety | 3 | 0.9 |  |
| Elevated transaminases | 3 | 0.9 |  |
| Fracture | 3 | 0.9 | YES (1 pt) |
| Gastroenteritis | 3 | 0.9 |  |
| Paresthesia/dysesthesia | 3 | 0.9 |  |
| Renal colic | 3 | 0.9 |  |
| Thrombocytosis | 3 | 0.9 |  |
| Urinary tract infection | 3 | 0.9 | YES (1 pt) |
| Bronchitis | 2 | 0.6 |  |
| Dental abscess | 2 | 0.6 |  |
| Hyperuricemia | 2 | 0.6 |  |
| Post-vaccine reaction | 2 | 0.6 |  |
| Acute hepatitis with cholestatic pattern compatible with toxic–drug-related etiology | 1 | 0.3 | YES (1 pt) |
| Biliary tract stones (hospitalized for obstructive jaundice due to biliary tract stones) | 1 | 0.3 |  |
| Blood spotting | 1 | 0.3 |  |
| Crusted lesions of the nasal cavities | 1 | 0.3 |  |
| Elevated bilirubin | 1 | 0.3 |  |
| Elevated LDH | 1 | 0.3 |  |
| Hemorrhoidal congestion | 1 | 0.3 |  |
| Hypotension | 1 | 0.3 |  |
| Immediate reaction to luspatercept (general malaise) | 1 | 0.3 |  |
| Low-grade evening fever of unclear origin and headache | 1 | 0.3 | YES (1 pt) |
| Myalgia | 1 | 0.3 |  |
| Nasal herpes simplex | 1 | 0.3 |  |
| Nausea | 1 | 0.3 |  |
| Near-syncope / faintness | 1 | 0.3 |  |
| Neoplasm | 1 | 0.3 | YES (1 pt) |
| Nocturnal hyperglycemia | 1 | 0.3 |  |
| OGTT diagnostic for diabetes | 1 | 0.3 |  |
| Pneumonia | 1 | 0.3 |  |
| Post-infectious colitis / enterocolitis | 1 | 0.3 | YES (1 pt) |
| Probable viral infection, thrombocytopenia, anemia | 1 | 0.3 | YES (1 pt) |
| Reduced ejection fraction | 1 | 0.3 | YES (1 pt) |
| Sepsis | 1 | 0.3 | YES (1 pt) |
| Severe dehydration, deferasirox toxicity | 1 | 0.3 | YES (1 pt) |
| Suspicious liver nodule | 1 | 0.3 |  |
| Transfusion reaction | 1 | 0.3 |  |
| Widespread pain | 1 | 0.3 |  |
| *Calculated as cases reported divided by total number of events reported.; multiple events for patients were considered.  **^§^** Other causes of hospitalization: see details in Table S7 | | | |

**Table S4** Variation of iron chelation therapy (ICT) during the period of observation

| **ICT variation by type** | **Cases(n=31)** |
| --- | --- |
| Variation of dose* | 18 |
| Variation of dose* & chelator | 4 |
| Variation of chelator | 9 |
| * Increase of dose n=5, decrease of dose n=7 |  |
| **ICT variation by reason** |  |
| Decrease in ferritin levels | 8 |
| MRI improvement | 3 |
| Decrease in ferritin levels & MRI improvement | 4 |

**Table S5** Association of transfusion independence (n=14) with patients’ characteristics

|  | **Univariate*** | | **Multivariate**** |  |
| --- | --- | --- | --- | --- |
|  | OR (CI 95%) | P | OR (CI 95%) | P |
| Transfusion burden^#^(>6 units vs ≤6 units) | 0.08 (0.02, 0.35) | <0.001 | 0.07 (0.01, 0.43) | 0.006 |
| Genotype (others vs β⁰/β⁰)^##^ | 16.7 (4.05, 114) | <0.001 | 17.9 (3.77, 151) | 0.001 |
| Serum ferritin (ng/ml) | 0.56 (0.30, 0.97) | 0.047 |  |  |
| Heart MRI-T2* (ms) | 1.08 (1.01, 1.19) | 0.054 |  |  |
| Age at diagnosis (year) | 1.07 (1.00, 1.16) | 0.056 |  |  |
| Age at the therapy start (year) | 1.06 (1.00, 1.13) | 0.066 |  |  |
| Hypogonadism (Yes vs No) | 0.33 (0.07, 1.15) | 0.11 |  |  |
| Estrogen-progestin therapy (Yes vs No) | 0.24 (0.01, 1.36) | 0.2 |  |  |
| White blood cells (10^9/L) | 2.6 (0.65, 11.4) | 0.2 |  |  |
| Smoke (Yes vs No) | 0.25 (0.01, 1.38) | 0.2 |  |  |
| Obesity (Yes vs No) | 5.15 (0.20, 136) | 0.3 |  |  |
| History of deep vein thrombosis (Yes vs No) | 5.15 (0.20, 136) | 0.3 |  |  |
| Left ventricular ejection fraction (LVEF) below 56% (Yes vs No) | 0.36 (0.02, 2.08) | 0.3 |  |  |
| AST (U/L) | 0.97 (0.90, 1.02) | 0.3 |  |  |
| Splenectomy (Yes vs No) | 1.62 (0.50, 5.24) | 0.4 |  |  |
| Creatinine (mg/dL) | 0.33 (0.02, 0.83) | 0.4 |  |  |
| LIC (mg/g dw) | 0.97 (0.88, 1.04) | 0.4 |  |  |
| Age at first transfusion (years) | 1.03 (0.94, 1.09) | 0.5 |  |  |
|  |  |  |  |  |
| *Only variable with a percentage of completion >70% and a significance level of association >0.5 were reported.  **Multivariate logistic regression model was generated using a stepwise forward regression approach based on the Akaike Information Criterion (AIC), and considering variables with an overall percentage of completion >90%, completed for responders and association with responders with a level of significance p < 0.20 at the univariate analysis. The variable and ferritin and white blood cells were log-transformed to improve normality of the distributions.  ^#^Transfusion burden in the period 12-weeks period before start luspatercept  ^##^other genotype:  β⁰/β^+^, β^+^/β^+^,HbE/β^+^ | | | | |
|  |  | |  | |

**Table S6** Adverse events of particular interest: Thromboembolic events.

|  | **Pt #1**  **DVT and PE** | **Pt #2**  **DVT and PE** | **Pt #3**  **SVT** |
| --- | --- | --- | --- |
| Sex | M | F | M |
| Beta globin genotype | β0/β+ | β0/β0 | - |
| Age at time of the event (years) | 39 | 33 | 42 |
| Age at first transfusion (years) | 8 | 1.8 | 1.5 |
| Days of starting therapy | 218 | 312 | 10 |
| Splenectomy | YES | NO | YES |
| Median Hb before TEE finding (g/dL) | 9.9 | 10.1 | 9 |
| Antiplatelets/Anticoagulants before the event | NO | NO | NO |
| Platelets at baseline | 553 | 319 | 728 |
| Obesity | NO | NO | YES |
| Smoke | NO | YES | NO |
| Estro-progestinic therapy | NO | YES* | NO |
| Congenital thrombophilia |  | YES | YES |
| Family history of thrombophilia | NO | - | YES |
| Other risk factors | NO | NO | YES** |
| Drug interruption | YES | YES | YES |
| *Contraceptive purposes  **Previous HCV; Hypothyroidism; Previous sepsis  DVT: Deep Venous Thrombosis; PE: Pulmonary Embolism; TEE: ThromboEmbolic Events | | | |

**Table S 7** Adverse events of particular interest: change in the Extramedullary Hematopoiesis Masses status

|  | **Pt#A** | **Pt#B** |
| --- | --- | --- |
| Location | - | Paravertebral |
| Newly developed EMH | YES | NO |
| Symptoms | NO |  |
| Median Hb before EMH finding | 9.6 | 9.7 |
| Days of starting therapy | 857 | 796 |
| Age at the time of the event | 21.3 | 52.2 |
| Sex | M | F |
| Age at first transfusion | 1.1 | NA |
| Beta globin Genotype | β0/β0 | β0/β0 |
| Splenectomy | NO | NO |
| Drug interruption | NO | NO |
| Other treatments | NO | NO |
| EMH: Extramedullary Hematopoiesis Masses | | |

**Table S 8** Causes of hospitalization (details) occurred in ‘compassionate’ phase of Luspatercept and in the first 12 follow-up in the post-marketing phase.

| **Hospitalization cases (n=25, 17 pts)** | **Cases (n)** |
| --- | --- |
| Heart failure in secondary hemochromatosis with cardiac iron overload - fever of unclear origin | 1 |
| Acute heart failure in a patient with cardiac hemosiderosis | 1 |
| Heart failure and atrial fibrillation | 1 |
| Atrial fibrillation* | 7 |
| Palpitations and dyspnea during flu-like symptoms; no intervention required | 1 |
| Scheduled transcatheter ablation for paroxysmal atrial fibrillation | 1 |
| Acute hepatitis with cholestatic pattern compatible with toxic–drug-related etiology | 1 |
| Sepsi | 1 |
| Severe dehydration, deferasirox toxicity | 1 |
| Low-grade evening fever of unclear origin, headache | 1 |
| Fracture (right femur) | 1 |
| Hepatic resection of segments 5–6 for small-duct cholangiocarcinoma, moderately differentiated, pt1a G2 N0 | 1 |
| Obstructive jaundice due to biliary tract stones | 1 |
| Post-infectious enterocolitis | 1 |
| Probable viral infection, thrombocytopenia, anemia | 1 |
| Right limb pseudoarthrosis surgery | 1 |
| Surgery for right pseudoarthrosis: debridement of the pseudoarthrosis focus, fragment reduction, autologous bone graft, and fixation with HC screw | 1 |
| Urinary tract infection (admitted to ER) | 1 |
| Physiotherapy | 1 |
| *One case was a new onset |  |
